# Functional Ingredients from Agri-Food Waste: Effect of Inclusion Thereof on Phenolic Compound Content and Bioaccessibility in Bakery Products

**DOI:** 10.3390/antiox9121216

**Published:** 2020-12-02

**Authors:** Valentina Melini, Francesca Melini, Francesca Luziatelli, Maurizio Ruzzi

**Affiliations:** 1 CREA Research Centre for Food and Nutrition, Via Ardeatina 546, I-00178 Rome, Italy; francesca.melini@crea.gov.it; 2 DIBAF, University of Tuscia, Via C. de Lellis snc, I-01100 Viterbo, Italy; f.luziatelli@unitus.it (F.L.); ruzzi@unitus.it (M.R.)

**Keywords:** food waste, phenolic compounds, antioxidant capacity, bread, functional foods, bioavailability

## Abstract

Reducing food loss and waste is among the efforts to relieve the pressure on natural resources and move towards more sustainable food systems. Alternative pathways of food waste management include valorization of by-products as a source of phenolic compounds for formulation of functional foods. Bakery products may act as an optimal carrier of phenolic compounds upon fortification. The aim of this paper is to present and discuss the effect that the inclusion of functional ingredients from agri-food waste can have on phenolic content and bioaccessibility in bakery products. To this aim, methods for the recovery of phenolic compounds from agri-food waste are presented, and fortification of bakery products by waste from fruits, vegetables, and seed crops is discussed. Bioaccessibility studies on fortified food products are considered to identify gaps and needs in developing sustainable healthy foods. Fruit and vegetable by-products are among the food wastes mostly valorized as functional ingredients in bakery product formulation. Agri-food waste inclusion level has shown to correlate positively with the increase in phenolic content and antioxidant capacity. Nevertheless, further studies are required to assess bioaccessibility and bioavailability of phenolic compounds in enriched food products to estimate the potential of agri-food waste in promoting human health and well-being.

## 1. Introduction

FAO estimated that 1.3 billion tons of food, about one-third of the annual production for human use, is globally lost or wasted every year [1]. Food loss and waste equal a major loss of earth resources, such as land, water, and energy, and lead to greater greenhouse gas emissions, so as to contribute to climate change. Hence, reducing food loss and waste is among the efforts to relieve the pressure on natural resources and move towards more sustainable food systems. Within the 2030 Agenda for Sustainable Development, the United Nations Member States set the target of halving per capita global food waste by adopting specific measures to reduce food losses along production and supply chains, including post-harvest losses (Target 12.3) [2].

Industrial processing and households are the major contributors to food waste. Campaigns have been put in place to reduce food waste at the household level, while several strategies have been identified to reduce food waste from industrial processing and manufacturing. Conventional management of food waste encompasses production of renewable energy, animal feeds, and compost. Alternative pathways include the valorization of food waste as a source of bioactive compounds to be used as functional food ingredients or nutraceuticals [3].

Waste from the industrial processing of plant-based products has been reported as a valuable source of bioactive molecules, such as phenolic compounds (PCs) [4]. These components play a crucial role in the prevention of non-communicable diseases, such as cardiovascular pathologies, type 2 diabetes, some types of cancer, and neurodegenerative diseases [5]. The intake of phenolic compounds is hence part of healthy diets.

Recently, the need to move towards healthy diets from sustainable food systems has been stressed, so as to nurture human health and foster environmental sustainability [6]. The enrichment of food products that are part of the dietary tradition, by using functional ingredients from agri-food waste (AFW), is thus a step into meeting this need.

Cereal-based products are major staples consumed worldwide on a daily basis by people of all ages and from all walks of life. In addition, they have advantages, such as ease of consumption and palatability. They are an important source of energy and nutrients, such as carbohydrate, protein, B group vitamins, and minerals, but are not a carrier of phenolic compounds when they are formulated with refined flours, because these components are lost during grain processing [7].

The enrichment of cereal-based products by AFW may thus have positive implications from environmental, economic, and health points of view. The addition of powders and extracts from AFW to pasta in order to increase the content in phenolic compounds has been recently reported [8]. As regards bakery products, enrichment by AFW commonly has a negative impact on technological and sensory quality. Lower volume, increased hardness, different color, and extraneous flavors can be observed [9]. Hence, only small quantities of AFW can be used. So far, the effect of AFW inclusion in the formulation of bakery products has been reviewed by focusing on macro- and micro-nutrients [10], while the contribution of AFW to phenolic compounds content and antioxidant capacity has been disregarded.

The aim of this paper is to present and critically discuss the effect that the inclusion of functional ingredients from AFW can have on the nutritional quality of enriched bakery products by focusing on phenolic compound content and bioaccessibility thereof. The determination of bioaccessibility and bioavailability of bioactive molecules is in fact a crucial aspect in evaluating the their bioefficacy and in assessing the effectiveness of food enrichment. To this aim, methods for the recovery of PCs from AFW are first presented; fortification of bakery products by waste from fruits, vegetables, and seed crops is discussed; and bioaccessibility studies on enriched food products are taken into consideration to identify gaps and needs in developing sustainable healthy foods.

## 2. Dietary Phenolic Compounds: Classification, Biotransformation, and Health Benefits

PCs are a diverse group of secondary metabolites synthesized by plants in normal and stress conditions [11]. They include a diversity of chemical structures sharing the presence of one or more hydroxyl groups on the aromatic ring(s). Based on their carbon skeleton structure, they can be classified into different groups ranging from simple phenols to complex polyphenols. Phenolic acids, flavonoids, and tannins are the most important dietary PCs.

**Figure 1:**
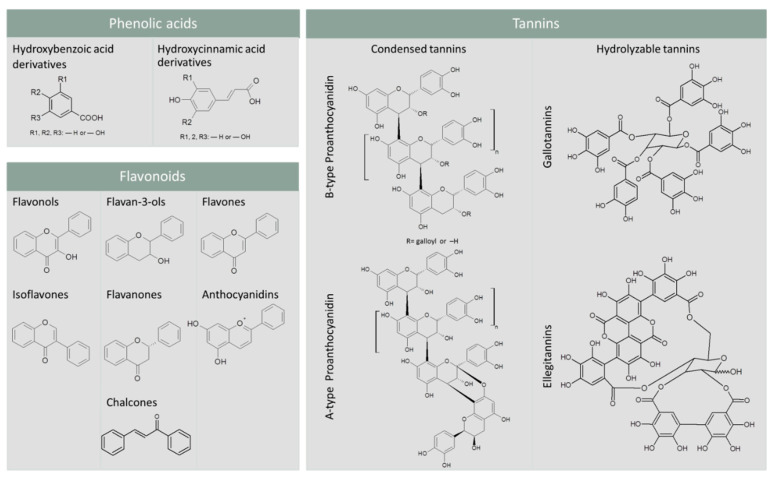
Skeleton structure of the main dietary phenolic compounds.

Phenolic acids are the predominant PCs in plant foods and are commonly found in grains, legumes, and oilseeds. They include hydroxybenzoic acid (HBA) and hydroxycinnamic acid (HCA) derivatives (Figure 1). In plant foods, HBA derivatives are mainly found as components of hydrolysable tannins and lignin, while HCA derivatives mostly occur as hydroxyacid esters that are covalently conjugated to the plant cell wall components (e.g., polysaccharides, lignin, glycoproteins, polyamines, and hydroxy fatty acids) [12].

Flavonoids account for about 60% of the total dietary PCs [12]. They are composed of a three-ring structure in the C6–C3–C6 form. Commonly, two phenolic rings are connected through a three-carbon “bridge” included in a six-member heterocyclic ring. Based on the connection of an aromatic ring to the heterocyclic ring, or on the oxidation state and functional groups of the heterocyclic ring, flavonoids can be further classified into flavonols, flavan-3-ols, flavones, isoflavones, flavanones, anthocyanidins, and chalcones [13] (Figure 1).

Tannins vary from dimers to large polymers and include condensed tannins, also known as proanthocyanidins, hydrolyzable tannins, complex tannins, and phlorotannins. Condensed and hydrolyzable tannins are the two main classes [14]. The former are oligomers or polymers composed of flavan-3-ol units. They can differ by their mean degree of polymerization, by the type of linkage, and the type of substitution patterns, namely galloylation and hydroxylation [14]. Depending on the interflavanic bond between the subunits, condensed tannins can be differentiated into B-type or A-type. In the former, flavan-3-ol units are linked by C4-C8 or C4-C6 bonds. A-type proanthocyanidins differ from B-type proanthocyanidins by at least one C=C linkage and an additional ether bond between the carbon-2 of one unit and the carbon-7 (C2-O-C7) of the adjacent unit (Figure 1). Hydrolyzable tannins can be further subgrouped into gallotannins and ellagitannins, which respectively yield gallic and ellagic acids, upon hydrolysis (Figure 1) [15].

Dietary PCs have beneficial effects on human health thanks to their antioxidant capacity. They act as antioxidants by preventing transition metal-mediated formation of hydroxyl free radicals. Actually, the oxidative stress caused by reactive oxygen species plays a pivotal role in the onset of atherosclerosis, neoplasia, and neurodegenerative diseases [16,17,18].

It has been reported that dietary intake of PCs contributes to preventing cardiovascular diseases and various cancers [5]. They also protect against the onset of neurodegenerative diseases [19].

**Figure 2:**
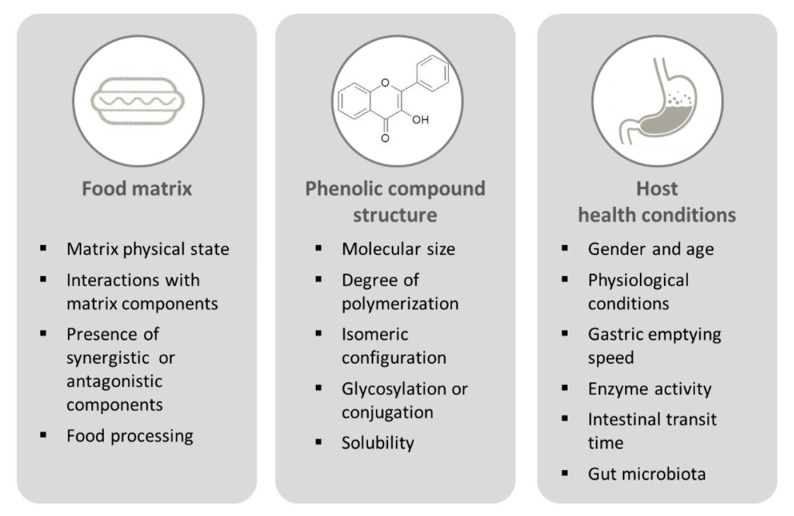
Factors affecting phenolic compound bioaccessibility and bioavailability.

In order to exert a beneficial effect, PCs need to reach a specific target organ or tissue. The key step in ensuring the bioefficacy of PCs is their bioavailability. From a nutritional perspective, bioavailability is the amount of any bioactive compound (or nutrient) that becomes available for normal physiological functions or storage, after absorption by the gut [20]. The first step for a bioactive compound to reach the target tissue/organ is its release from the food matrix. The fraction of a bioactive compound released from the food matrix in the gastrointestinal lumen and made available for intestinal absorption is referred to as bioaccessibility [20]. Bioaccessibility and bioavailability are affected by several factors, related to food matrix characteristics, phenolic compound structure, and host health conditions (Figure 2).

## 3. Functional Ingredients from Agri-Food Waste: Recovery of Phenolic Compounds

AFW originates throughout the whole food supply chain, from production to post-harvesting, industrial processing, distribution, domestic processing, and consumption, with wastage volumes differing among phases and food commodities.

In Europe, households contribute the most to food waste, with a share of 53%, followed by processing, which accounts for 19% of total food waste. The remaining 28% comes from food service (12%), production (11%), and wholesale and retail (5%) [21]. 

Each commodity group generates specific by-products. Fruit and vegetables produce great amounts of peelings, pomace, trimmings, seeds, stones, stems, and leaves [22]. Cereal grain milling generates bran, which accounts for 3–30% of the kernel weight on a dry basis, hulls, husks (4–14%), germ, broken grains (6–13%), and powders (7–12%) [23]. The legume industry produces great amounts of husks, pods, and off-quality grains [22]. Hulls, husks, skins, shells, and shattered cotyledons are the main waste of primary processing of nuts and oilseeds [23]. 

Within the current bioeconomy and sustainability framework, alternative handling of these by-products encompasses the recovery of bioactive molecules, including PCs.

Drying and size reduction techniques, extraction methods, and fermentation are the main strategies to turn AFW into functional ingredients.

### 3.1. Drying and Size Reduction Techniques

Food powders and flours are the simplest form into which AFW can be processed to be incorporated as a functional ingredient into conventional foods. The unit operations to get food powders and flours from AFW generally depend on the form of waste, which can be either liquid, solid, or a paste. In case of liquid waste, powders and flours are produced by applying a drying technique, while in case of a solid material, size reduction by crushing and grinding, milling, pulverization, granulation, and mixing must be applied [24]. Other factors affecting the choice of AFW handling methods are the heterogeneity and the structural differences of the waste, the coexistence of edible/non-edible parts [25], the shelf-life, and the necessity to preserve compounds of nutritional interest or with antioxidant properties. 

Waste from fruit, vegetable, and oilseed processing, such as pomace, commonly undergoes first drying, then size reduction. Conventional hot-air convection drying, low-temperature vacuum drying, freeze-drying, or microwave drying are among the conventional techniques applied to reduce water content in AFW. However, the choice of the drying method must be cautious, because application of high temperatures and/or presence of oxygen may degrade thermolabile compounds or molecules sensitive to oxidation. For instance, PCs may be degraded during air-drying due to polyphenol oxydase activity [26]. The effect of drying techniques on the bioactive compound content in AFW was studied, and drying parameters were optimized.

Michalska et al. investigated the effect of freeze-drying (FD), convective drying (CD; 50–90 °C), microwave vacuum (MWV; 120, 240, 360 and 480 W), and combination thereof on total polyphenol content in blackcurrant pomace [27]. It emerged that FD determined a decrease in total polyphenols; upon CD, a linear decrease in total polyphenol content occurred at increasing temperature, except for drying at 50 °C, possibly due to inactivation of polyphenol oxidase. When MWV drying was applied, a lower degradation of polyphenolic compounds was observed thanks to a shorter processing time.

Hot air (HA) and microwave-assisted hot air (MWHA) drying were applied in combination with extrusion to bilberry (*Vaccinium myrtillus* L.) press cake [28]. It was observed that MWHA drying allowed a moisture content of 17 g/100 g to be obtained in a shorter time (215 min) than HA (about 360 min); however, the phenolic compound content was not significantly different.

FD and oven-drying were applied to skins from two grape varieties and the effect on phenolic compound, anthocyanin, and flavonol content was investigated. FD enabled a higher preservation of bioactive molecules [29].

Different combinations of drying temperatures and times were tested on blueberry and grape pomace in order to preserve procyanidins and anthocyanins [30]. It was found that a temperature of 40 °C in a forced-convection oven did not affect the bioactive molecule content, drying at 60 °C caused a reduction in anthocyanins, while at 125 °C a significant loss (about 52%) was observed.

Some additional drying techniques (e.g., hot air convective drying, microwave vacuum drying, intermittent microwave convective drying, industrial rotary drying, freeze drying, radiofrequency, osmotic agents, etc.) have been so far applied in food processing [31,32,33,34,35,36,37], but their applicability in AFW drying and their effect on phenolic compound retention has not been yet explored. 

The particle size of AFW also affects the recovery of PCs. For example, a reduction of black currant pomace particle size from 0.5–1 to <0.125 mm determined a 1.6–5-fold increase in PCs [38]. Particle size reduction prior to drying also affected the content in phenolic compounds in waste from carrots and white cabbage [25]. As to carrot residues, the chopping (≤10 mm) and grinding (≤5 mm) pre-treatments did not significantly influence the bioactive molecule content, while chopped samples of white cabbage residue better preserved the phenolic compounds.

### 3.2. Extraction Methods

Novel environmental-friendly methods, including ultrasound-assisted extraction (UAE), microwave-assisted extraction (MAE), and supercritical fluid extraction (SFE), have been developed for sustainable recovery of PCs from AFW [4,39]. In addition, a new generation of sustainable solvents (i.e., deep eutectic solvents, DES) has been used.

UAE is based on the application of ultrasounds, which promote a greater diffusion of solvent into cellular materials, and thus improve mass transfer and cell wall disruption, so as to facilitate the release of bioactive components [39]. UAE prevents temperature increase and thermal degradation of bioactive compounds. It also allows a reduction of the extraction time, using lower quantities of solvent, cutting process costs, and benefiting from high-level automation [40,41]. UAE has been applied to the extraction of PCs from waste of the winemaking industry: anthocyanins from red grape pomace [42,43] and wine lees [44], trans-resveratrol from red grape waste [45], and polyphenols from red grape pomace [42,46]. As regards the supply chain of fruit and vegetables, UAE was applied to recover PCs from apple [47], *Vaccinium* berry [48], citrus [46,49,50], tomato [51], and onion wastes [52]. PCs were extracted by UAE also from olive waste (e.g., cake, leaves) [46,51]. The application of UAE to extraction of PCs from root and tuber wastes (e.g., potato peels), and (black) carrot pomace was also reported [53,54]. Among cereals and legumes, polyphenolic compounds were recovered by UAE from wheat bran [46] and mung bean hulls [55].

MAE is an extraction technique combining microwave and traditional solvent extraction. The principle that MAE is based on is dielectric heating, which consists in microwave electromagnetic radiation heating a dielectric material by molecular dipole rotation of the polar components present in the matrix [4]. Shorter extraction time, higher extraction rate, minor solvent requirements, higher selectivity towards added-value compounds, and lower costs over traditional extraction methods are some of the advantages that make MAE a favorable method in the extraction of bioactive compounds [4,39,40]. MAE has so far been used to recover different classes of PCs. Anthocyanins were recovered from grape juice [56], red grape [43], and black carrot wastes [57]. Polyphenols were extracted by MAE from red grape pomace [43] and red wine lees [58]. Flavonols were obtained from red grape waste. Hydroxytyrosol was recovered by MAE from olive pomace [59].

SFE is based on the use of fluids at pressure and temperature values above or near their critical points. In particular, SFE uses renewable solvents, such as CO_2_, and offers some advantages, such as easy recovery, selectivity, compound stability, reduced time, and an overall total energy saving. Apart from the unnecessity of solvent removal from the final product, the degradation process of bioactive compounds is also lower because of light and air absence [60]. SFE is especially suitable to recover extracts from solid matrices [4,39,40]. It has been applied to extract PCs from pomace of grape, apple, and orange [60,61,62] and from black walnut husks and hazelnut waste [60,63].

DES extraction is one more analytical approach to recover PCs from AFW. DESs are prepared by mixing two or more components (e.g., hydrogen bond acceptors or hydrogen bond donors) able to interact by hydrogen bonds. Compounds used for DES preparation comprise choline chloride, DL-malic acid, citric acid, glycerol, D-(+)-glucose, D-(−)-fructose, sucrose, D-(+)-galactose, D-(+)-maltose, maltitol, and D-(−)-ribose. DES exhibit the same extraction properties than organic solvents but have negligible environmental and economic impact. Moreover, they have a GRAS (generally recognized as safe) status. They have been applied to the extraction of several classes of PCs from grape waste: phenolic acids from grape skins and red grape pomace [64,65]; anthocyanin pigment derivatives from grape waste [44,64,65], and flavonol glycosides from red grape pomace [44]. DES were also used to extract PCs from onion waste [51,66]. Natural DES have been applied to extract polyphenols from olive pomace, in combination with homogenization, microwaves, ultrasounds, and high hydrostatic pressure [67], and from olive leaves, kernels, and cake [51,68,69].

### 3.3. Fermentation and Enzymatic Treatments

Bioprocesses, such as fermentation and enzyme technology, are further approaches for the transformation of AFW into value-added products. 

Solid-state fermentation (SSF) and sub-merged fermentation have mostly been used [70]. SSF by *Rhizopus oligosporus* and *Aspergillus niger* was applied to apricot pomace. The use of *R. oligosporus* as a starter determined an increase in the total phenolic content (TPC) by 70% and in the total flavonoid content (TFC) by 38%. SSF by *A. niger* increased TPC by more than 30% and TFC by 12% [71]. SSF with *A. niger* was also applied to pomace from black and dwarf elderberries, and an increase in extractable phenolics by 11.11% and 18.82% was observed, respectively [72]. SSF was also applied to grape pomace, and the production of xylanase allowed the release of PCs from the substrate [73].

As regards the application of SSF to cereal by-products, *A. niger* was used to ferment wheat bran, and higher PC content was obtained thanks to the activity of β-glucosidase enzymes [73]. Wheat bran was also fermented by a strain of *Aspergillus oryzae* and the TPC of ethanolic and methanolic extracts was higher in fermented samples than in the non-fermented ones [74]. The application of a strain of *Lactobacillus brevis* and *Candida humilis* to wheat bran enabled the release of PCs, thanks to the activity of cell wall-degrading enzymes [75].

Enzyme-assisted extraction (EAE) of PCs from AFW has also been reported [40]. It is based on the ability of enzymes, such as cellulase, β-glucosidase, xylanase, β-gluconase, and pectinase, to degrade cell wall structure and depolymerize plant cell wall polysaccharides, so as to prompt the release of bound compounds [76]. The specificity of enzymes for their substrate also determines an increase in the bioactivity of extracts, thanks to the hydrolysis of high-molecular-weight compounds to a lower molecular weight [77]. The use of water as a solvent in EAE, instead of organic chemicals, also makes EAE an eco-friendly technology for extraction of bioactive compounds. Applications of EAE to extraction of PCs from grape waste, pistachio green hulls, and pomegranate peels have been reported [78,79,80].

## 4. Agri-Food Waste Contribution to Phenolic Compound Content and Antioxidant Capacity in Bakery Products

Powders, flours, or extracts from AFW have recently been used as functional ingredients in formulation of bakery products, in order to improve their nutritional value, in terms of phenolic compound content and antioxidant capacity.

Fruit and vegetables, and seed and oilseed crops are the main waste commodity groups from which functional ingredients for bakery product formulation have been obtained so far.

### 4.1. Fortification of Bakery Products by Functional Ingredients from Fruit and Vegetable Waste

Fruit and vegetables are mostly consumed as fresh foods, playing a primary role in healthy diets. When processed, significant amounts of peels, skin, seeds, stems, leaves, pomace, and scraps are obtained. Their transformation into powders, flours, or extracts to be used in bakery product formulation is among the valorization strategies of AFW.

#### 4.1.1. Fruit Waste

The fortification of bakery products by functional ingredients from by-products of mango, orange, and pomegranate has been recently explored [81,82,83].

Massive quantities of peels, seeds/kernels, and bagasse, still containing nutrients and bioactive compounds, are obtained from the industrial processing of mango (*Mangifera indica* L.) [23]. These wastes have been converted into powders and flours, and used as functional ingredients. Raw and defatted flours from mango seed kernels were used as wheat flour substituents in bread-making at five different substitution levels (from 5 to 25% *w/w*) [81]. It was found that the higher the percentage of mango seed kernel flour was, the higher TPC was. In bread added with raw mango seed kernel flour, TPC ranged between 91.13 and 128.35 mg Gallic Acid Equivalents (GAE) 100 g^−1^ dry matter (DM) (Table 1). When defatted mango seed kernel flour was used with a substitution level ranging between 10 and 25% *w/w*, TPC was significantly higher than the control, with values ranging between 91.71 and 106.74 mg GAE 100 g^−1^ DM.

The antioxidant capacity was also determined since it is generally correlated to the content in PCs. It emerged that, when raw mango seed kernel flour was added, bread showed higher antioxidant capacity than the control by using both the DPPH and FRAP methods, except for 5% (*w/w*) mango seed kernel flour bread, which had an antioxidant capacity DPPH value comparable to the control [81]. The antioxidant capacity values in bread added with defatted mango kernel flour, determined by the DPPH assay, were higher than the control at a substitution level between 15% and 25% (*w/w*). The FRAP assay showed higher antioxidant capacity values at higher substitution levels of wheat flour. Several factors, such as the mango variety, the ripening stage at harvesting, and the post-harvest handling, can affect the content of PCs in mango by-products. Moreover, the conditions applied to the preparation of functional ingredients from food waste may influence the quantitative and qualitative composition of functional ingredients. All these aspects should be considered in order to maximize the nutraceutical potential of enriched food products.

As regards citrus fruits, about one-third of the total production is processed into juices, jams, and jellies [23]. According to FAO, the production of citrus fruits, including oranges, lemons and limes, and tangerines, in 2018 was higher than 129 million tons [84]. Hence, massive quantities of by-products are commonly obtained. Peels and seeds are the main waste. The former are rich in PCs, such as flavonoids and hydroxycinnamates [23], hence their inclusion in bakery product formulation has been investigated and the effect on the nutritional value of enriched products has been determined. The formulation of pearl millet biscuits with four levels of citrus waste flours has been recently investigated, and TPC, TFC, and antioxidant capacity were determined [82]. In orange peel-added biscuits, TPC ranged between 832 and 1187 mg GAE 100 g^−1^ DM. TFC varied from 334 to 812 mg Quercetin Equivalents (QE) 100 g^−1^ DM. These values were significantly higher than the control. Antioxidant capacity values, determined by the ABTS method, ranged between 148 and 219 µM Trolox Equivalents Antioxidant Capacity (TEAC) 100 g^−1^ DM. No significant differences (*p* < 0.05) were found between samples with 15% and 20% (*w/w*) addition of orange peel flour. The inhibition of DPPH radicals was found to increase with the addition of orange peel flour in biscuits, as well.

Pomegranate seeds are the main waste from the pomegranate juice industry. They contain oil rich in conjugated fatty acids, high-quality proteins, dietary fiber, and PCs [85]. Gluten-free (GF) bread enriched with pomegranate seed powder at substitution levels between 2.5 and 10% (*w/w*) was made [83], and TPC and antioxidant capacity were determined. In pomegranate seed powder-added bread, TPC ranged between 129 and 247 mg GAE 100 g^−1^ DM, while in the control, it was 88 mg GAE 100 g^−1^ DM. As regards antioxidant capacity, DPPH and ABTS assays were used, and results were expressed as EC_50_, which is the maximum concentration required to obtain a 50% antioxidant effect. The lower the EC_50_ values are, the higher the antioxidant capacity is. It was found that all enriched GF bread samples showed higher activities than the control, except for 2.5% (*w/w*) substituted bread, whose DPPH value was not statistically different from the control. The highest antioxidant capacity for DPPH was observed in bread with 7.5% (*w/w*) of pomegranate seed powder, while for ABTS, the maximum activity was found for bread with 10% (*w/w*) of substitution.

As regards waste from grape processing, the main by-product is marc, which is the bulk of skins, pulp, seeds, and stems of the fruit left over after wine or juice production. The use of grape marc in the formulation of cereal-based products has been widely explored [86,87,88,89]. Most research studies investigated the use of grape marc in the fortification of pasta, and only Pasqualone et al. explored the addition of grape marc extracts in the formulation of durum wheat biscuits [86]. Grape marc extract was used as a water substituent in the dough preparation, and the ratio between durum semolina and grape marc extract was 2:1 g mL^−1^. TPC in enriched biscuits was approximately 1.4-fold higher than the control. TFC was 3.1 mg Catechin Equivalents (CE) 100 g^−1^ DM in the control and increased to 48.1 mg CE 100 g^−1^ DM in enriched biscuits. In addition, it emerged that grape marc extract-added biscuits also contained anthocyanins and proanthocyanidins that were not detected in the control sample. In AFW-enriched products, the antioxidant capacity was approximately 1.5-fold higher than in the control. 

#### 4.1.2. Vegetable Waste

Unit operations, such as washing, sorting, trimming, peeling, coring, cutting, slicing, and dicing, targeted to transform raw vegetables into canned, frozen, ready-to-cook, and ready-to-eat products, generate considerable amounts of both solid and liquid wastes. Fresh-cut and ready-to-cook products currently available on the market comprise lettuce, broccoli, onions, carrots, tomatoes, spinach, and leafy greens, among others.

During the preparation of fresh-cut salad, the external leaves and the core are discarded, while they might be further processed and converted into functional ingredients. Recently, Plazzotta et al. demonstrated that flour obtained from lettuce wastes can be used in bread formulation [90]. It was found that the addition of lettuce waste flour significantly increased TPC, compared to the control (43.55 mg GAE 100 g^−1^ DM) (Table 1). In enriched bread, TPC ranged between 58.56 and 340.62 mg GAE 100 g^−1^ DM. The higher the lettuce waste inclusion level was, the higher TPC was. The antioxidant capacity was 3873.3 OD^−3^ min^−1^ kg^−1^ DM in control bread, while in enriched bread, values ranged between 4486.7 and 10,290.0 OD^−3^ min^−1^ kg^−1^ DM.

Broccoli are among the most widely produced vegetables worldwide, alongside tomatoes, cucumbers and gherkins, chilies and peppers, cauliflowers, pumpkins, squash, and gourds [23]. Considerable amounts of broccoli leaves are discarded at harvesting. Moreover, the industrial dissection of broccoli produces a combination of florets and stalks as residues, while several by-products, such as stems, mixtures of leaves and inflorescences, blanched inflorescence, and stems, are generated during the production of frozen broccoli. These by-products can be further processed into powders whose use as functional ingredients in bakery product formulation has been tested. A flour from broccoli stems and leaves has been incorporated into bread and GF bakery product dough [91,92]. Lafarga et al. formulated two wheat breads including 2% (*w/w*) powder obtained from either leaves or stems [91]. Both broccoli by-products have been reported to have high content in PCs and high antioxidant and anticancer activities [93]. In enriched bread, TPC was 8–11% higher than in control bread, with values ranging from 184 to 189 mg GAE 100 g^−1^ DM. No significant (*p* < 0.05) difference was found in bread added with powder from broccoli leaves or stems. Regardless of the analytical method, the antioxidant capacity of both AFW-enriched bread types was significantly higher than in the control. In bread obtained by using broccoli leave powder, the antioxidant capacity was 2.31 or 4.4 mg Ascorbic Acid Equivalents (AAE) 100 g^−1^ DM based on the DPPH or FRAP method, respectively. In broccoli stem powder-added bread, the antioxidant capacity, measured by the DPPH and FRAP method, was 2.25 and 4.6 mg AAE 100 g^−1^ DM, respectively.

Drabińska et al. reported on the formulation of GF mini sponge cakes added with broccoli leaves processed into a fine powder and added at three different substitution levels, ranging between 2.5 and 7.5% *w/w* [92]. It emerged that TPC in enriched GF mini sponge cakes was higher than the control and the increase ranged between 67% and 115%. The higher the addition of broccoli leave powder was, the higher TPC was. At the 7.5% substitution level, TPC was approximately 1 mg GAE g^−1^ DM. The antioxidant capacity of GF mini sponge cakes increased in a dose-dependent manner with broccoli leave powder content. In enriched products, the antioxidant capacity measured by ABTS or FRAP was 257 to 419 and 104 to 277 μmol TEAC 100 g^−1^ DM, respectively.

In contrast with most vegetable waste, which can be recycled as feed or fertilizers, onion by-products represent a serious environmental issue, since they are not suitable for fodder due to their aroma, nor can they be used as fertilizers because of the rapid developments of phytogenetic agents [94]. Great amounts of waste from onions are produced from the industrial processing, including preparation of dehydrated onions in the form of flakes and powder, or canned onion pickle, as well as from onions that do not meet the quality standards for marketing. Onion skins, the two outer fleshy scales, the top and bottom of the bulbs, and damaged bulbs are the main waste [95].

A fine powder obtained from the apical trimmings of onion bulbs and the outer layers was used in the formulation of bread, at five different substitution levels ranging between 1 and 5% (*w/w*) [96]. It was observed that the higher the level of onion waste powder addition was, the higher TPC, TFC, and antioxidant capacity were. TPC values ranged between 62 and 164 mg GAE 100 g^−1^ DM. However, the difference in TPC between bread at 4% and 5% of onion powder was not statistically significant. TFC varied from 26 to 168 mg QE 100 g^−1^ DM. Antioxidant capacity determined by the DPPH or FRAP methods was 1.00 to 2.81 and 2.12 to 5.41 mM TEAC g^−1^ DM, respectively. The highest value of antioxidant capacity by DPPH was obtained for bread at 4% (*w/w*) onion powder addition, while by FRAP, it was obtained for bread at the 5% substitution level. However, the sensory evaluation analysis showed that bread with a satisfactory acceptability to consumers was obtained up to a 3% onion powder addition.

Besides powders, extracts of PCs can be obtained from onion skins and used as functional ingredients in bread-making. Piechowiak et al. formulated bread by the addition of dried onion skin extract at a concentration between 0.1 and 0.5% (*w/w*) [97]. The effect of supplementation on TPC and antioxidant capacity was investigated. TPC in enriched bread ranged between 30 and 75 mg GAE 100 g^−1^ DM. The higher the onion extract addition was, the higher TPC was. Antioxidant capacity determined by the DPPH and CUPRAC methods was up to 14- and 7-fold higher in enriched bread than in control bread, respectively. DPPH-measured antioxidant capacity varied between 30 and 325 mg TEAC 100 g^−1^ DM; CUPRAC-measured antioxidant capacity ranged between 30 and 185 mg TEAC 100 g^−1^ DM.

Generally, a partial substitution of flour with fruit and vegetable wastes results in an increase in TPC and antioxidant capacity, hence value-added products can be obtained. However, the sensory quality of enriched food products must be investigated since the replacement can have negative effects on the product sensory attributes and limit their appraisal by consumers. Moreover, the bioaccessibility and bioavailability of PCs should be assessed in order to estimate the nutraceutical potential of enriched products.

### 4.2. Fortification of Bakery Products by Functional Ingredients from Seed and Oilseed Crop Waste

The main seed crops for human consumption include cereals, pseudocereals, legumes, nuts, and oilseeds.

Cereal grains are commonly processed by dry or wet milling in order to obtain refined cereals or milling fractions to be used as ingredients in the formulation of cereal products. The milling process also produces several by-products [98], including cereal bran (CB), whose annual production amounts to around 150 million tons [99]. CB has been used as a food ingredient in the production of bakery products. Its inclusion has mostly been targeted to increase the content in dietary fiber. However, CB is also rich in bioactive molecules, including PCs. Bran extracts have been used in pasta-making, and higher TPC in supplemented pasta was observed [100]. The improvement by SSF of the phenolic content and profile of wheat and oat bran has been studied [101]; however, to the best of our knowledge, their use for the enrichment of bakery products has not been explored. Alongside bran, wheat germ is a major by-product of wheat milling. Despite being rich in bioactive components, it has been rarely used in food formulation due to the lipid content, which makes it subjected to rancidity [98].

In the agro-industrial processing of legumes (e.g., canning, freezing, and/or drying), a mixture of leaves, stems, empty pods, and dark or spotted seeds are obtained. Residues represent about 5–25% of the harvested crop [102]. Legume by-products have been mainly used to recover functional ingredients, such as proteins, and to a lesser extent, dietary fiber and PCs.

Flours from carob by-products and extracts from soybean and chickpea husks have been used as functional ingredients in bread-making [103,104]. Bread enriched with flours from carob by-products (e.g., germ, pods, and seed peels) showed a TPC higher than the control [103] (Table 2). Carob pod and carob germ flour addition enabled the highest TPC to be obtained. As regards antioxidant capacity, no statistically significant differences were observed between the addition of carob germ and carob pod flour.

Supplementation of wheat bread by extracts from soybean or chickpea by-products was also investigated [104]. The addition of soybean extract allowed a 4.4-fold increase of TPC, while in chickpea husk extract-added bread, TPC was 4.7-fold higher than the control. Antioxidant capacity was measured by the DPPH assay, ABTS, and FRAP methods. In bread enriched with soybean extract, a 3-fold (DPPH assay) and 2-fold (ABTS and FRAP methods) increase were observed, with respect to the control. In chickpea extract-added bread, the antioxidant capacity was 1.5- to 4-fold higher than in the control.

As regards nut primary seed processing, hulls and husks are the main waste [23]. They are commonly discarded or recycled for animal feeding. Nevertheless, they still contain value-added molecules, such as antioxidants, dietary fiber, minerals, vitamins, and proteins. The use of powder from nut waste as functional ingredients has been explored in some bakery products. Velioǧlu et al. reported on the inclusion of hazelnut testa, a by-product obtained from hazelnut roasting, in the formulation of bread, cookies, and cakes [105]. Different inclusion levels, ranging from 4 to 10% (*w/w*), were tested in bread formulation. It emerged that TPC ranged between 669.0 and 1942.7 mg GAE 100 g^−1^ DM in enriched bread, and the highest values were obtained at the higher inclusion level (Table 2). As regards hazelnut testa-enriched cookies, TPC was 3- to 6-fold higher than the control. A significant increase in TPC was also observed when hazelnut testa was used in cake preparation. The higher the inclusion level was, the higher TPC was.

The production of coffee also produces considerable amounts of by-products, such as cherry husks, cherry pulps, silver skin, and spent coffee [106]. GF bread enriched with extracts from coffee husk or silver skin was formulated by Guglielmetti et al. [107]. In bread made with extracts from coffee husk and silver skin, TPC was 121.12 and 254.92 mg chlorogenic acid (CGA) g^−1^ DM, respectively, while in the control bread, it was 54.69 mg CGA g^−1^. A 3.78- and 1.70-fold increase of antioxidant capacity was observed in bread enriched with extracts (Table 2), possibly due to the occurrence of chlorogenic acid and melanoidins. The content of chlorogenic acid was 2 mg 100 g^−1^ in bread with coffee husk and 25 mg 100 g^−1^ in the formulation with silver skin extract.

Cocoa processing mainly generates three types of waste: cocoa pod husk, cocoa bean shells, and cocoa mucilage [108]. Their valorization includes their use as a source of bioactive compounds, namely dietary fiber, antioxidants, and theobromine [108]. Cocoa husk was used in the formulation of extruded and non-extruded corn snacks, at three different addition levels (5%, 10%, and 15%) [109], and the effect of inclusion on TPC and antioxidant capacity was evaluated. It was observed that TPC and antioxidant capacity in enriched snacks were higher than the control and increased proportionally with the addition of cocoa husks. However, in extruded products, TPC and the antioxidant capacity were lower than in non-extruded ones (Table 2).

As to oilseeds, they are mainly processed into edible oils and several by-products are produced. Hulls are generated from the dehulling step, which is followed by seed grinding and heating to completely destruct oil cells. Then, oilseeds undergo mechanical pressing or direct solvent extraction, and oil cake (or pomace) is obtained [39]. Water waste is also obtained from olive oil production. These by-products are rich in phenolic acids, flavonoids, and tannins. They can be, thus, used as functional ingredients or as a source of bioactive compounds in food formulation and fortification.

Chia seed pomace, obtained from the pressing of chia seed oil, was used in the formulation of both gluten-containing (GC) and GF bread [110]. A 5% (*w/w*) substitution of wheat flour with chia seed pomace at 6% and 15% of fat content was explored. It emerged that in chia seed pomace-added GC bread, TPC was approximately 3% higher than in the control, and no statistically significant (*p* < 0.05) differences were found in the use of 6% and 15% fat chia seed pomace. The antioxidant capacity increased from 28% (control GC bread) to 35% in both enriched GC bread (Table 2). A similar trend was observed in the formulation of GF bread added with chia seed pomace at both 6% and 15% of fat content. TPC level increased from approximately 30 (control bread) to 35–36 mg GAE 100 g^−1^ DM in enriched GF bread. The antioxidant capacity was 33% in control GF bread and 40% in enriched GF bread.

The use of wastes from olive oil production in bread and bread-substitute formulation has been extensively investigated [111,112,113,114,115,116]. 

Olive paste (OP) was used in bread formulation by Cedola et al. [111,112]. In bread formulated with 10% dry OP flour, TPC was 7-fold higher than in the control (28 mg GAE 100 g^−1^ DM), and TFC was 14-fold higher (6 mg QE 100 g^−1^). The antioxidant capacity was 0.24 mg TEAC g^−1^ DM in the control sample, and increased to 21.64 mg TEAC g^−1^ DM in OP-added bread [111] (Table 2). Olive mill waste water (OMWW), OP, and a combination of OP and OMWW were also used [112]. Among the tested functional ingredients, the combination of OMWW and OP enabled the highest TPC (180 mg GAE 100 g^−1^ DM) to be obtained. As far as the antioxidant capacity is concerned, the highest values were observed in bread formulated with OMWW+OP. In detail, the antioxidant capacity in OMWW+OP bread was approximately 8- and 13-fold higher than the control by using the ABTS and FRAP methods, respectively. Durante et al. formulated “taralli”, an Italian traditional bakery product used as a bread substitute or a savory snack, by substitution of durum semolina with 20% (*w/w*) of fermented OP from the olive cultivars Cellina di Nardò and Leccino [113]. It was found that TPC, calculated as the sum of HPLC data, was 1377 µg g^−1^ DM in “taralli” obtained with OP from Cellina di Nardò and 1016 µg g^−1^ DM in Leccino OP-added “taralli” (Table 2). In the control, phenolic compounds were not detected by chromatographic analysis. Hydroxytyrosol was the main polyphenol compound, accounting for 66% and 77% of total polyphenols in “taralli” with Cellina di Nardò and Leccino OP, respectively. “Taralli” enriched with extracts from olive leaves, a waste of olive mills, were formulated by Cedola et al. [114]. Olive leaf extract substituted white wine, which is a traditional ingredient for “taralli” production. In cooked enriched “taralli”, TPC was 61 mg GAE/100 g DM, while in the control, it was 43 mg GAE/100 g DM. With respect to the control, a 4-fold increase in TFC was also observed (i.e., 36 mg QE/100 g DM vs. 9 mg QE/100 g DM). The antioxidant capacity in enriched “taralli” was 4.86 µmol FeSO_4_ 7H_2_O/g.

Olive pomace (OPM) from virgin olive oil production was used to formulate bread and other cereal-based products, such as granola bars and pasta [115]. In control bread, no PCs were detected, while in OPM-added bread, the hydroxytyrosol content was 235 µg g^−1^ DM and tyrosol was 43 µg g^−1^ DM (Table 2). The recovery of PCs with respect to OPM was 49%.

The use of OPM in bread-making and the effect on phenolic compound content was also studied by Di Nunzio et al. [116]. Whole wheat flour was replaced by OPM (4% *w/w*) and two different fermentation methods were used: baker’s yeast and sourdough. In enriched bread, TPC was 115% (baker’s yeast fermented) and 206% (sourdough fermented) higher than the corresponding control. In addition, it was observed that in enriched bread fermented by baker’s yeast TPC was higher than in sourdough fermented bread (876.7 and 617.2 mg/kg DM, respectively), possibly because microbial enzymes degrade the cell wall structure during fermentation so as to promote the release of the aglycones linked to fibers. Di Nunzio et al. also formulated whole einkorn flour biscuits with 2.5% dry OPM. The level of OPM addition was based on the limit of organoleptic acceptance assessed by a consumer preference test. In enriched biscuits, TPC was approximately 40% higher than the control [116].

## 5. Bioaccessibility and Bioavailability of Phenolic Compounds in Bakery Products Enriched with Agri-Food Waste

A high dietary intake of PCs does not entail an equivalent bioactivity thereof. As a matter of fact, bioactivity is strictly related to the bioaccessibility and bioavailability of the ingested molecule [20]. Hence, the evaluation of bioaccessibility and bioavailability is mandatory to evaluate the efficacy of efforts made to increase the content of PCs in food.

Despite the high number of studies assessing the phenolic compound content in food products formulated with functional ingredients from AFW, only a few investigated the bioaccessibility and bioavailability of these bioactive components.

Lafarga et al. applied an in vitro gastrointestinal digestion to bread enriched with flours from broccoli by-products in order to study the phenolic compound resistance to digestion and the total antioxidant capacity (TAC) [91]. As regards bread with flour from broccoli stems, TPC increased by 66% after gastric digestion and 164% after the intestinal step. In bread with flour from broccoli leaves, a 106% increase in TPC was found after the gastric stage and 170% after the intestinal stage. In both bread formulations, a significant increase of TAC was also observed, with respect to the pre-digestion stage (Table 3).

The bioaccessibility of phenolic compounds in GF bread samples formulated with extracts from coffee husk and silver skin was studied and compared to the bioaccessibility of control bread [107]. No significant differences were found between digested GF bread added with coffee husk extract and the control. It emerged also that TPC in the soluble fraction of silver skin extract-added bread was significantly higher than the control, even after digestion (265.70 vs. 227.92 CGA g^−1^, respectively). The antioxidant capacity of each enriched bread was significantly higher than the control (Table 3).

Cedola et al. reported that enrichment of bread with dry olive paste flour (10%) enabled an increase of the bioaccessibility of PCs by 10% [111]. They observed that the total polyphenols were stable in the enriched bread under gastric and small-intestinal digestive conditions.

The same research group also investigated the bioaccessibility of phenolic compounds in “taralli” formulated with olive-leaf extract, as a substituting ingredient for white wine [114]. They found that TPC, TFC, and antioxidant capacity of olive-leaf extract-formulated “taralli” were higher after digestion. They also investigated the content of the three most abundant polyphenols present in olive leaf extracts (i.e., oleuropein, hydroxytyrosol, and verbascoside) at the three stages of the digestion process, by HPLC. They found that oleuropein was slightly resistant to gastric digestion, while hydroxytyrosol and verbascoside were sensitive to the process. Moreover, oleuropein was nearly completely degraded in the intestinal phase (Table 3).

Colantuono et al. investigated the bioaccessibility of PCs in bread enriched with artichoke stem powder at three different concentrations [117]. They found that the level of polyphenols released from the enriched bread was correlated with the concentration of artichoke stem powder used in the bread formulation. For all substitution levels, about 80% of polyphenols were released in the duodenal phase, and phenolic acids were the most abundant. The remaining fraction of totally bioaccessible compounds was released in the colon step. Hence, the health effects of the enriched bread may be mediated by the intestinal microbiota.

## 6. Conclusions and Future Perspectives

The use of AFW as a source of functional ingredients for the formulation of bakery products has been greatly studied. Among food waste categories, fruit and vegetable by-products have been mostly explored to increase TPC in bakery products. AFW powders and flours are the form of choice for inclusion in bakery products.

Experimental data on AFW-enriched bakery products showed that the level of inclusion correlates positively with the increase in TPC and antioxidant capacity. However, studies were mainly focused on the determination of TPC, while it would be relevant to determine the phenolic profile, as well. In addition, studies mainly investigated the content in PCs upon substitution of wheat flour with powders, while the estimation of PC bioaccessibility and bioavailability has so far been overlooked. Efforts should be made to assess the bioaccessibility and bioavailability of PCs in enriched food products in order to reliably estimate the effectiveness of fortification and the potential of AFW in promoting human health and well-being, and to understand if, by a cascading effect, reducing food waste will contribute to achieving other Sustainable Development Goals, such as the Zero Hunger goal (SDG 2). It was also observed that most studies on the experimental formulation of bakery products with AFW did not consider the sensory profile and the safety of produced food products. Insights into these aspects might by far complete the knowledge on this issue and on the possibility to scale up the use of AFW.

## Figures and Tables

**Table 1:** TPC and antioxidant capacity of cereal-based products formulated by the addition of waste from fruit and vegetables.

Agri-Food Waste (AFW)	Functional Ingredient from AFW	AFW-Enriched Product	TPC (mg GAE 100 g^−1^ DM) and TFC (mg QE 100 g^−1^ DM)	Antioxidant Capacity	Reference
Mango seed kernel (raw)	Powder/Flour	Bread added with raw mango seed kernel flour at a substitution level of 5, 10, 15, 20, and 25% (*w/w*)	TPC (control): 85.00 TPC (5%): 91.13 * TPC (10%): 100.15 TPC (15%): 112.59 TPC (20%): 119.70 TPC (25%): 128.35	DPPH (control bread): 24.35 ^1^ DPPH (5%): 27.35 ^1,^* DPPH (10%): 29.71 ^1^ DPPH (15%): 33.17 ^1^ DPPH (20%): 37.82 ^1^ DPPH (25%): 41.57 ^1^ FRAP (control bread): 372.6 ^2^ FRAP (5%): 387.4 ^2^ FRAP (10%): 398.6 ^2^ FRAP (15%): 412.4 ^2^ FRAP (20%): 426.9 ^2^ FRAP (25%): 441.4 ^2^	Amin et al. [81]
Mango seed kernel (processed defatted)	Powder/Flour	Bread added with processed defatted mango kernel flour at a substitution level of 5, 10, 15, 20, and 25% (*w/w*)	TPC (control): 85.00 TPC (5%): 88.39 * TPC (10%): 91.71 TPC (15%): 95.42 TPC (20%): 99.44 TPC (25%): 106.74	DPPH (control bread): 24.35 ^1^ DPPH (5%): 25.06 ^1,^* DPPH (10%): 27.46 ^1,^* DPPH (15%): 29.53 ^1^ DPPH (20%): 33.49 ^1^ DPPH (25%): 36.39 ^1^ FRAP (control bread): 372.6 ^2^ FRAP (5%): 379.3 ^2^ FRAP (10%): 387.8 ^2^ FRAP (15%): 395.8 ^2^ FRAP (20%): 405.5 ^2^ FRAP (25%): 420.1 ^2^	Amin et al. [81]
Orange peel	Powder/Flour	Biscuits added with orange peel flour at a substitution level of 5, 10, 15, and 20% (*w/w*)	TPC (control): 584 TPC (5%): 832 TPC (10%): 960 TPC (15%): 1032 TPC (20%): 1187 TFC (control): 120 TFC (5%): 334 TFC (10%): 627 TFC (15%): 784 TFC (20%): 812	ABTS (control): 117 ^3^ ABTS (5%): 148 ^3^ ABTS (10%): 206 ^3^ ABTS (15%): 219 ^3^ ABTS (20%): 219 ^3^ DPPH (control): 238 ^4^ DPPH (5%): 211 ^4^ DPPH (10%): 197 ^4^ DPPH (15%): 192 ^4^ DPPH (20%): 171 ^4^	Obafaye et al. [82]
Pomegranate seeds	Powder/Flour	GF bread added with pomegranate seed powder at a substitution level of 2.5, 5, 7.5, and 10% (*w/w*)	TPC (control): 88 TPC (2.5%): 129 TPC (5%): 143 TPC (7.5%): 216 TPC (10%): 247	DPPH (control): 25.97 ^5^ DPPH (2.5%): 29.39 ^5,^* DPPH (5%): 13.55 ^5^ DPPH (7.5%): 14.24 ^5^ DPPH (10%):11.97 ^5^ ABTS (control): 9.95 ^5^ ABTS (2.5%): 6.22 ^5^ ABTS (5%l): 5.99 ^5^ ABTS (7.5%): 5.16 ^5^ ABTS (10%): 6.14 ^5^	Bourekoua et al. [83]
Grape marc	Extract	Biscuits added with grape marc extract (flour:grape marc extract ratio ≈ 2:1 g/mL)	TPC (control): 44 TPC (enriched): 62.9 TFC (control): 3.1 mg CE 100 g^−1^ DM TFC (enriched): 48.1 mg CE 100 g^−1^ DM Total anthocyanins (control): nd Total anthocyanins (enriched): 140 mg Mv-3-glc_equ_ kg^−1^ Proanthocyanidins (control): nd Proanthocyanidins (enriched): 151 mg Cy-Cl_equ_ kg^−1^	DPPH (control): 33.2 ^1^ DPPH (enriched): 48.1 ^1^ ABTS (control): 472 μmol TEAC kg^−1^ DM ABTS (enriched): 790 μmol TEAC kg^−1^ DM	Pasqualone et al. [86]
Lettuce waste	Powder/Flour	Wheat bread added with lettuce waste flour at a substitution level of 2, 4, 12, and 40% (*w/w*)	TPC (control): 43.55 TPC (2%): 58.56 TPC (4%): 73.12 TPC (12%): 135.45 TPC (20%): 340.62	DPPH (control): 3873.3 ^6^ DPPH (2%): 4486.7 ^6^ DPPH (4%): 4644.4 ^6^ DPPH (12%): 5602.2 ^6^ DPPH (20%): 10290.0 ^6^	Plazzotta et al. [90]
Broccoli stems or leaves	Powder/Flour	Bread added with broccoli by-product powder (stems or leaves) at a substitution level of 2% (*w/w*)	TPC (control): ≈ 170 TPC (enriched, stems): ≈ 189 TPC (enriched, leaves): ≈184	FRAP (control): ≈ 3.9 ^7^ FRAP (enriched, stems): ≈ 4.6 ^7^ FRAP (enriched, leaves):≈ 4.4 ^7^ DPPH (control): ≈ 1.65 ^7^ DPPH (enriched, stems):≈ 2.25 ^7^ DPPH (enriched, leaves):≈ 2.31 ^7^	Lafarga et al. [91]
Broccoli leaves	Powder/Flour	GF mini sponge cakes added with broccoli leaf powder at a substitution level of 2.5, 5, and 7.5% (*w/w*)	TPC (control): 46 TPC (2.5%): 77 TPC (5%): 87 TPC (7.5%): 99	ABTS (control): 115 ^3^ ABTS (2.5%): 257 ^3^ ABTS (5%): 322 ^3^ ABTS (7.5%): 419 ^3^ FRAP (control): 24 ^3^ FRAP (2.5%): 104 ^3^ FRAP (5%): 173 ^3^ FRAP (7.5%): 277 ^3^	Drabińska et al. [92]
Onion (apical trimmings of the bulbs, and the outer dry and semidry layers)	Powder/Flour	Wheat bread added with industrial onion waste at a substitution level of 1, 2, 3, 4, and 5% (*w/w*)	TPC (control): 49 TPC (1%): 62 TPC (2%): 102 TPC (3%): 124 TPC (4%): 158 TPC (5%): 164 TFC (control): nd TFC (1%): 26 TFC (2%): 57 TFC (3%): 76 TFC (4%): 138 TFC (5%): 168	DPPH (control): 0.16 ^8^ DPPH (1%): 1.00 ^8^ DPPH (2%): 1.49 ^8^ DPPH (3%): 2.08 ^8^ DPPH (4%): 2.81 ^8^ DPPH (5%): 2.66 ^8^ FRAP (control): 0.70 ^8^ FRAP (1%): 2.12 ^8^ FRAP (2%): 3.40 ^8^ FRAP (3%): 4.33 ^8^ FRAP (4%): 5.27 ^8^ FRAP (5%): 5.41 ^8^	Prokopov et al. [96]
Onion skin	Extract	Bread added with dried onion skin extract at a substitution level of 0.1, 0.25, and 0.5% (*w/w*)	TPC (control): ≈ 12 TPC (0.1%): ≈ 30 TPC (0.25%): ≈ 55 TPC (0.5%): ≈ 75	DPPH (control): ≈ 23 ^9^ DPPH (0.1%): ≈ 30 ^9^ DPPH (0.25%): ≈ 100 ^9^ DPPH (0.5%): ≈ 325 ^9^ CUPRAC (control): ≈ 24 ^9^ CUPRAC (0.1%): ≈ 30 ^9^ CUPRAC (0.25%): ≈ 110 ^9^ CUPRAC (0.5%): ≈ 185 ^9^	Piechowiak et al. [97]

^1^ DPPH % of inhibition; ^2^ µmol 100 g^−1^ DM; ^3^ µM TEAC 100 g^−1^ DM; ^4^ mg 100 mL^−1^ DM; ^5^ EC_50_ mg mL^−1^ DM; ^6^ OD^−3^ min^−1^ kg^−1^ DM; ^7^ mg AAE 100 g^−1^ DM; ^8^ mM TEAC g^−1^ DM; ^9^ mg TEAC 100 g^−1^ DM; * not statistically different from the control; TPC: total phenolic content; TFC: total flavonoid content; DM: dry matter; TEAC: Trolox Equivalents Antioxidant Capacity; AAE: ascorbic acid equivalents; GAE: gallic acid equivalents; nd: not detected; QE: quercetin equivalents; CE: catechin equivalents; Mv-3-glc_equ_: malvidin 3-O-β-D-glucoside equivalents; Cy-Cl_equ_: cyanidin chloride equivalents.

**Table 2:** TPC and antioxidant capacity of bakery products formulated by the addition of waste from seed and oilseed crops.

Agri-Food Waste (AFW)	Functional Ingredient from AFW	AFW-Enriched Product	TPC and TFC	Antioxidant Capacity	Reference
Carob germ	Powder/Flour	Bread added with carob germ flour (8% *w/w*)	TPC (control): 1.73 ^1^ TPC (enriched): 5.53 ^1^	DPPH (control): 3.77 ^5^ DPPH (enriched): 26.45 ^5^ TEAC (control): 11.45 ^6^ TEAC (enriched): 25.84 ^6^ FRAP (control): 0.06 ^7^ FRAP (enriched): 0.24 ^7^ ORAC (control): 4.59 ^6^ ORAC (enriched): 26.41 ^6^	Rico et al. [103]
Carob pod	Powder/Flour	Bread added with carob pod flour (8% *w/w*)	TPC (control): 1.73 ^1^ TPC (enriched): 5.95 ^1^	DPPH (control): 3.77 ^5^ DPPH (enriched): 22.16 ^5^ TEAC (control): 11.45 ^6^ TEAC (enriched): 26.05 ^6^ FRAP (control): 0.06 ^7^ FRAP (enriched): 0.27 ^7^ ORAC (control): 4.59 ^6^ ORAC (enriched): 29.56 ^6^	Rico et al. [103]
Carob seed peel	Powder/Flour	Bread added with carob seed peel flour (8% *w/w*)	TPC (control): 1.73 ^1^ TPC (enriched): 2.32 ^1^	DPPH (control): 3.77 ^5^ DPPH (enriched): 18.19 ^5^ TEAC (control): 11.45 ^6^ TEAC (enriched): 12.04 ^6,^* FRAP (control): 0.06 ^7^ FRAP (enriched): 0.09 ^7,^* ORAC (control): 4.59 ^6^ ORAC (enriched): 21.40 ^6^1	Rico et al. [103]
Soybean and chickpea husk	Extract	White bread added with soybean or chickpea husk extract (2% *w/w*)	TPC (control): 23.2 ^2^ TPC (enriched, soybean): 103.6 ^2^ TPC (enriched, chickpea): 110.1 ^2^	DPPH (control): 0.354 ^6^ DPPH (enriched, soybean): 1.096 ^6^ DPPH (enriched, chickpea): 1.167 ^6^ ABTS (enriched, control): 1.145 ^6^ ABTS (enriched, soybean): 2.567 ^6^ ABTS (enriched, chickpea): 3.035 ^6^ FRAP (enriched, control): 0.819 ^6^ FRAP (enriched, soybean): 1.800 ^6^ FRAP (enriched, chickpea): 1.247 ^6^	Niño-Medina et al. [104]
Hazelnut testa	Powder/Flour	Bread added with hazelnut testa at a substitution level of 4, 6, 8, and 10% *w/w*	TPC (control bread): 205.7 ^2^ TPC (4%):669.0 ^2^ TPC (6%): 1058.1 ^2^ TPC (8%): 1499.1 ^2^ TPC (10%): 1942.7 ^2^	-	Velioǧlu et al. [105]
Hazelnut testa	Powder/Flour	Cookies added with hazelnut testa at a substitution level of 4, 6, 8, and 10% *w/w*	TPC (control): 156.8 ^2^ TPC (4%): 497.7 ^2^ TPC (6%): 654.5 ^2^ TPC (8%): 808.0 ^2^ TPC (10%): 977.7 ^2^	-	Velioǧlu et al. [105]
Hazelnut testa	Powder/Flour	Cake added with hazelnut testa at a substitution level of 4, 6, 8, and 10% *w/w*	TPC (control): 167.8 ^2^ TPC (4%): 645.7 ^2^ TPC (6%): 805.0 ^2^ TPC (8%): 1134.2 ^2^ TPC (10%): 1312.6 ^2^	-	Velioǧlu et al. [105]
Chia seed pomace	Powder/Flour	Wheat bread added with chia seed pomace (5% *w/w*) at 6 and 15% of fat content	TPC (control):≈ 23 ^2^ TPC (enriched, pomace 6% fat): ≈ 29 ^2^ TPC (enriched, pomace 15% fat): ≈ 28 ^2^	DPPH (control): 28 ^5^ DPPH (enriched, pomace 6% fat): 35 ^5^ DPPH (enriched, pomace 15% fat): 35 ^5^	Zdybel et al. [110]
Chia seed pomace	Powder/Flour	GF bread (maize and rice flour 1:1) with chia seed pomace (5% *w/w* substitution) at 6 and 15% of fat content	TPC (control):≈ 30 ^2^ TPC (enriched, pomace 6% fat): ≈ 36 ^2^ TPC (enriched, pomace 15% fat): ≈ 35 ^2^	DPPH (control): 33 ^5^ DPPH (enriched, pomace 6% fat): 40 ^5^ DPPH (enriched, pomace 15% fat): 40 ^5^	Zdybel et al. [110]
Coffee husk	Extract	GF bread added with coffee husk extract (2.5%)	TPC (control): 54.69 ^10^ TPC (enriched): 121.12 ^10^ Chlorogenic acid (control): nd Chlorogenic acid (enriched): 2 ^11^	TAC (control): 76.10 ^10^ TAC (enriched): 129.39 ^10^	Guglielmetti et al. [107]
Coffee silver skin	Extract	GF bread added with coffee silver skin extract (2.5%)	TPC (control): 54.69 ^10^ TPC (enriched): 254.92 ^10^ Chlorogenic acid (control): nd Chlorogenic acid (enriched): 25^11^	TAC (control): 76.10 ^10^ TAC (enriched): 288.27 ^10^	Guglielmetti et al. [107]
Cocoa husk	Powder/Flour	Corn snack enriched with cocoa husk (5%, 10% and 15%)	TPC (non-extruded, control): 55.17 ^2^ TPC (non-extruded, enriched 5%): 84.37 ^2^ TPC (non-extruded, enriched 10%): 105.14 ^2^ TPC (non-extruded, enriched 15%): 109.91 ^2^ TPC (extruded, control): 48.63 ^2^ TPC (extruded, enriched 5%): 72.25 ^2^ TPC (extruded, enriched 10%): 83.99 ^2^ TPC (extruded, enriched 15%): 105.68 ^2^	TAC (non-extruded, control): 11.03 ^5^ TAC (non-extruded, enriched 5%): 11.24 ^5^ TAC (non-extruded, enriched 10%): 19.60 ^5^ TAC (non-extruded, enriched 15%): 23.47 ^5^ TAC (extruded, control): 11.25 ^5^ TAC (extruded, enriched 5%): 20.55 ^5^ TAC (extruded, enriched 10%): 25.76 ^5^ TAC (extruded, enriched 15%): 33.08 ^5^	Jozinović et al. [109]
Dry olive paste	Powder/Flour	Bread enriched by dry olive paste flour (10% *w/w*)	TPC (control): 28 ^2^ TPC (enriched): 196 ^2^ TFC (control): 6 ^3^ TFC (enriched): 85 ^3^	ABTS (control): 0.24 ^8^ ABTS (enriched): 21.64 ^8^	Cedola et al. 2019 [111]
Olive mill waste water (OMWW) and olive paste (OP)	Water and Powder/Flour	Bread added with i) olive mill wastewater (OMWW) (3:5 water:flour *w/w*), ii) olive paste (OP) (10% *w/w*), and iii) OMWW+OP	TPC (control): 14 ^2^ TPC (OMWW): 49 ^2^ TPC (OP): 133 ^2^ TPC (OMWW +OP): 180 ^2^	ABTS (control): 0.046 ^8^ ABTS (OMWW): 0.08 ^8^ ABTS (OP): 0.42 ^8^ ABTS (OMWW +OP): 0.67 ^8^ FRAP (control): 1.8 ^7^ FRAP (OMWW): 5.6 ^7^ FRAP (OP): 17 ^9^ FRAP (OMWW +OP): 25.3 ^7^	Cedola et al. 2020 [112]
Olive paste	Powder/Flour	“*Taralli*” added with fermented olive paste (20% *w/w*) from black olives of cultivar Cellina di Nardò (CdN) or Leccino (LEC)	TPC (control): nd TPC (enriched, CdN): 1377 ^4^ TPC (enriched, LEC): 1016 ^4^	-	Durante et al. [113]
Olive-leaf extract	Extract	“*Taralli*” added with olive-leaf extract	TPC (control): 43 ^2^ TPC (enriched): 61 ^2^ TFC (control): 9 ^3^ TFC (enriched): 36 ^3^	FRAP (control): 3.48 ^9^ FRAP (enriched): 4.86 ^9^	Cedola et al. [114]
Olive pomace	Powder/Flour	Bread added with olive pomace (freeze-dried, 5% *w/w*)	Hydroxytyrosol (control): nd Hydroxytyrosol (enriched): 235 ^4^ Tyrosol (control): nd Tyrosol (enriched): 43 ^4^	-	Cecchi et al. [115]
Olive pomace	Powder/Flour	Whole wheat bread (baker’s yeast fermented or sourdough) added with defatted olive pomace (4% *w/w*)	Baker’s yeast fermented: TPC (control): 756.1 ^4^ TPC (enriched): 876.7 ^4^ Sourdough fermented: TPC (control): 299.6 ^4^ TPC (enriched): 617.2 ^4^	-	Di Nunzio et al. [116]
Olive pomace	Powder/Flour	Whole einkorn biscuits added with defatted olive pomace (2.5% *w/w*)	TPC (control): 226 ^4^ TPC (enriched): 316 ^4^	-	Di Nunzio et al. [116]

^1^ µmol GAE g^−1^ DM; ^2^ mg GAE 100 g^−1^ DM; ^3^ mg QE 100 g^−1^ DM; ^4^ µg g^−1^ DM; ^5^ DPPH % of inhibition; ^6^ µmol TEAC g^−1^ DM; ^7^ µmol Fe g^−1^ DM; ^8^ mg TEAC g^−1^ DM; ^9^ µmol FeSO_4_ 7H_2_O/g; ^10^ mg CGA g^−1^ DM; ^11^ mg 100 g^−1^; * not statistically different from the control; TPC: total phenolic content; TFC: total flavonoid content; GAE: gallic acid equivalents; nd: not detected; QE: quercetin equivalents.

**Table 3:** Studies on phenolic compounds bioaccessibility and bioavailability in bakery products added with agri-food waste.

Agri-Food Waste (AFW)	AFW-Enriched Product	In Vitro Method	Phenolic Compound Bioaccessibility/Bioavailability	Effect of Digestion on Antioxidant Capacity	Reference
Broccoli stems	Bread enriched with broccoli by-product flour	Three sequential stages including a simulated salivary fluid (α—amylase, pH 7.0), gastric (pepsin, pH 3.0), and intestinal (pancreatin and fresh bile, pH 7.0) phase	TPC: ↑ after gastric stage (66%) ↑ after intestinal stage (164%) compared to the pre-digestion stage.	TAC: ↑ after gastric stage (419%, FRAP and 96% DPPH) and intestinal stage (429% FRAP and 104% DPPH) compared to the pre-digestion stage	Lafarga et al. [91]
Broccoli leaves	Bread enriched with broccoli by-product flour	Three sequential stages including a simulated salivary fluid (α—amylase, pH 7.0), gastric (pepsin, pH 3.0), and intestinal (pancreatin and fresh bile, pH 7.0) phase	TPC: ↑ after gastric stage (106%) and intestinal stage (170%) compared to the pre-digestion stage	TAC: ↑ after gastric stage (540%, FRAP and 112% DPPH) and intestinal stage (655% FRAP and 227% DPPH) compared to the pre-digestion stage.	Lafarga et al. [91]
Coffee husk	GF bread added with coffee husk extract (2.5%)	Three sequential stages including a salivary step (pH 6.9, 5 min, 3.9 U/mL amylase, aerobic), a gastric step (pH 2, 90 min, 71.2 U/mL pepsin, aerobic), and duodenal step (pH 7, 150 min, 9.2 mg/mL pancreatin and 55.2 mg/mL bile extract, aerobic)	TPC (control, digested): 227.92 mg CGA g^−1^ TPC (enriched, digested): 222.18 mg CGA g^−1^ *	TAC (insoluble fractions): ↑ by 34% compared to the digested control	Guglielmetti et al. [107]
Coffee silver skin	GF bread added with coffee silver skin extract (2.5%)	Three sequential stages including a salivary step (pH 6.9, 5 min, 3.9 U/mL amylase , aerobic), a gastric step (pH 2, 90 min, 71.2 U/mL pepsin, aerobic), and duodenal step (pH 7, 150 min, 9.2 mg/mL pancreatin and 55.2 mg/mL bile extract, aerobic)	TPC (control, digested): 227.92 mg CGA g^−1^ TPC (enriched, digested): 265.70 mg CGA g^−1^	TAC (insoluble fractions): ↑ by 53% compared to the digested control	Guglielmetti et al. [107]
Olive oil paste	Bread enriched by dry olive paste flour (10% *w/w*)	Three-stage simulated digestion including oral, gastric and small intestinal phase	Bioaccessibility of polyphenols of digested bread with dry olive paste addition was ≈ 70% In digested control bread, bioaccessibility of polyphenols was 60%	-	Cedola et al. [111]
Olive-leaf extract	“Taralli” added with olive-leaf extract	Three-stage simulated digestion including oral, gastric and small intestinal phase	TPC (before digestion): 54 mg GAE/100 g TPC (after digestion): 323 mg GAE/100 g TFC (before digestion): 36 mg QE/100 g TFC (after digestion): 88 mg QE/100 g	TAC (before digestion): 4.86 µmol FeSO_4_ 7H_2_O/g TAC (after digestion): 20.98 µmol FeSO_4_ 7H_2_O/g	Cedola et al. [114]
Artichoke stem powder (ASP) at 3, 6 and 9% (*w/w*) substitution	Bread enriched with artichoke stem powder	Three sequential stages including a simulated salivary fluid (α--amylase), gastric phase (porcine pepsin, pH 3.0), and intestinal fluid (pancreatin and fresh bile)	TPs (3% ASP bread): 649.3 µg g^−1^ DM (after duodenal phase) 121.3 µg g^−1^ DM (after colon phase) TPs (6% ASP bread): 1423.0 µg g^−1^ DM (after duodenal phase) 321.8 µg g^−1^ DM (after colon phase) TPs (9% ASP bread): 1958.6 µg g^−1^ DM (after duodenal phase) 520.5 µg g^−1^ DM (after colon phase)	-	Colantuono et al. [117]

↑: increase; TAC: Total Antioxidant Capacity; TPs: Total Polyphenols; TPC: Total phenolic content; CAE: chlorogenic acid equivalents; * not statistically different from the control.
